# Enhancing canola breeding by editing a glucosinolate transporter gene lacking natural variation

**DOI:** 10.1093/plphys/kiac021

**Published:** 2022-01-25

**Authors:** Yizhou He, Zhiquan Yang, Minqiang Tang, Qing-Yong Yang, Yuanyuan Zhang, Shengyi Liu

**Affiliations:** The Key Laboratory of Biology and Genetic Improvement of Oil Crops, The Ministry of Agriculture and Rural Affairs of PRC, Oil Crops Research Institute, Chinese Academy of Agricultural Sciences, Wuhan 430062, China; National Key Laboratory of Crop Genetic Improvement, College of Informatics, Huazhong Agricultural University, Wuhan 430070, China; College of Forestry, Hainan University, Haikou 570228, China; National Key Laboratory of Crop Genetic Improvement, College of Informatics, Huazhong Agricultural University, Wuhan 430070, China; The Key Laboratory of Biology and Genetic Improvement of Oil Crops, The Ministry of Agriculture and Rural Affairs of PRC, Oil Crops Research Institute, Chinese Academy of Agricultural Sciences, Wuhan 430062, China; The Key Laboratory of Biology and Genetic Improvement of Oil Crops, The Ministry of Agriculture and Rural Affairs of PRC, Oil Crops Research Institute, Chinese Academy of Agricultural Sciences, Wuhan 430062, China

## Abstract

A low seed glucosinolate resource was developed in polyploid *B. napus* using a method that identifies the functions of genes with rare or no genetic variation.

Dear Editor,

A major challenge in current genetics study is to identify functions of genes with rare or no genetic variation through forward genetics approaches, such as quantitative trait locus mapping and association study in germplasm, and particularly in polyploid crops, it is difficult to study functional differentiation of duplicated genes. Here, we report a causal gene with rare mutation in glucosinolate transport and creation of a genotype of low seed glucosinolate for quality and resistance breeding of polyploid *Brassica napus* canola, the second largest global source of edible oil and protein meal. 

Glucosinolates are well-known secondary metabolites with important biological and economic roles in plant defense to disease and insects and human nutrition/health, such as anti-cancer effect (Sønderby et al., 2010). However, high seed meal glucosinolates can cause goiter and other harmful effects. Thus “double-low” (low seed glucosinolate and low erucic acid content) canola breeding was initiated in the mid-20th century, which has dramatically reduced seed glucosinolate content from .100 µmol·g^−1^ to <30 µmol·g^−1^ (Kondra and Stefansson, 1970). Unfortunately, glucosinolate content in vegetative tissues also decreased dramatically due to disruption of the biosynthesis pathway, which caused decreased plant disease resistance (Liu et al., 2020). Although the pathways of glucosinolate biosynthesis in vegetative tissues and subsequent transport to developing seeds have been well-characterized in *Arabidopsis thaliana* (Jørgensen et al., 2017) and mutation of the genes encoding glucosinolate transporters (GTRs) substantially reduced seed glucosinolates in *Brassica* plants (Nour-Eldin et al., 2012, 2017), low seed glucosinolate germplasms already created by gene-editing in *B. napus* cannot be applied for breeding because of their negative effects on other traits (Tan et al., 2022), probably due to involvement of edited genes in other trait formation.

To address the above problems, first we comprehensively identified all homologs of *A. thaliana GTRs* from 12 de novo assembled *B. napus* genomes (Chalhoub et al., 2014; Zou et al., 2019; Lee et al., 2020; Song et al., 2020; Supplemental Figure S1) and those from one high glucosinolate cultivar ZY821 were divided into three subclades showing sequence differentiation of *GTR1*, *GTR2*, and *GTR3* (Figure 1A). Then we detected expression patterns of all 20 *BnaGTRs* in 20 stages/tissues by RNA-seq from the representative *B. napus* accessions with high (cv. ZY821) and low (cv. ZS11) seed glucosinolate content. The expression patterns of these *BnaGTRs* are very complicated and there was no clear cue to judge their contribution to glucosinolates except for *BnaA06.GTR2* and *BnaA02.GTR2* showing higher expression in some tissues (Figure 1B; Supplemental Table S1). Subsequently, we performed a genome-wide association study on seed glucosinolate content of 312 diverse *B. napus* germplasm accessions collected worldwide (Supplemental Table S2). Based on single-nucleotide polymorphisms genotyped by whole-genome resequencing and aligned on ZY821 genome, three loci harboring five *BnaGTR2s* on chromosomes A02, A09, and C02 were significantly associated with seed glucosinolate content (Figure 1C). Surprisingly, four *BnaGTRs* located in significantly associated loci lowly or moderately expressed, while *BnaA06.GTR2* and *BnaC03.GTR2*, whose genomic regions did not show significant association, exhibited high expression in silique wall of late development stages (Figure 1B) and *BnaA06.GTR2* showed higher expression levels than those of *BnaC03.GTR2*.

**Figure 1:**
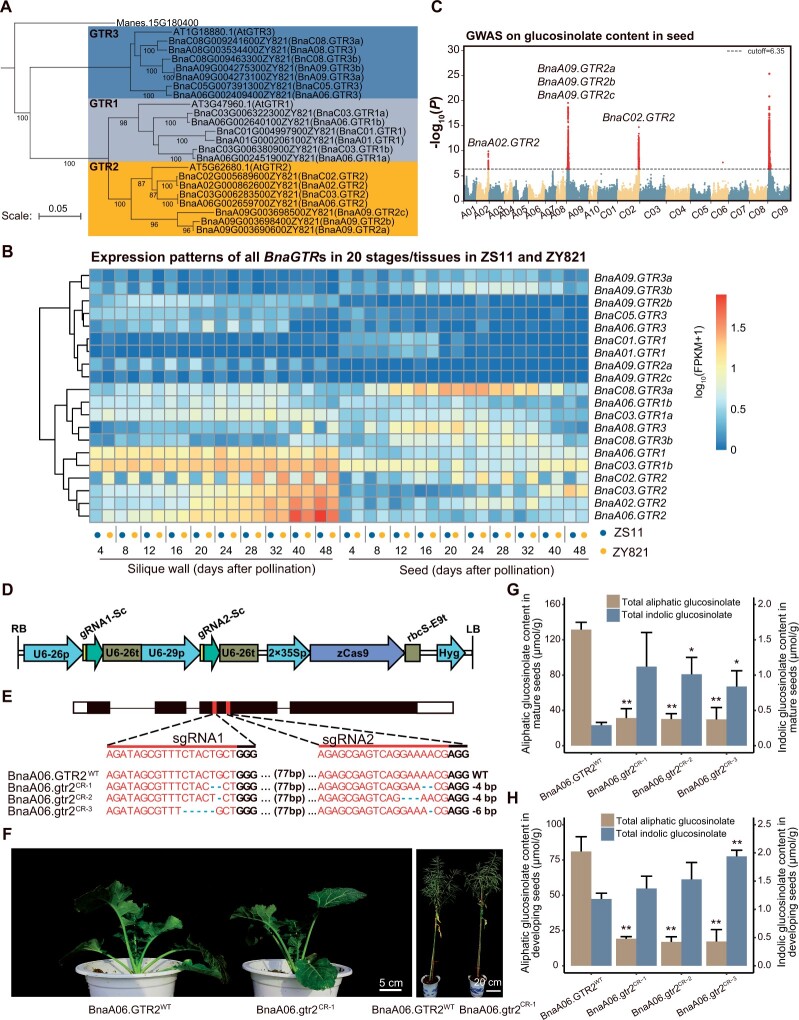
Identification of a glucosinolate transporter gene with rare natural mutation in *B. napus*. A, Neighbor-joining tree of GTRs homologs in *A. thaliana* and *B. napus*. MANIHOT ESCULENTA CYANOGENIC GLUCOSIDE TRANSPORTER-1 (MeCGTR1) was used as an out-group. Bootstrap values above 75% were given at branch nodes. The scale bar indicates the number of amino acid residue substitutions per site. B, Expression patterns of *BnaGTR2s* in the low (ZS11) and high (ZY821) seed glucosinolate accessions in the different tissues at the different growth stages. C, Manhattan plot of genome-wide association study on seed glucosinolate content. Horizontal dashed line represents the significant threshold (−log_10_(*P*) = 6.35). Red dots indicated the significantly associated single-nucleotide polymorphisms with seed glucosinolate content. D, Schematic of the constructed genome-editing vector with two gRNAs to target *BnaA06.GTR2* sequence. RB/LB, right/left border of T-DNA; U6-26p and U6-29p, two *A. thaliana U6* gene promoters; 35Sp, cauliflower mosaic virus 35S promoter; U6-26t, *A. thaliana U6* gene terminator; rbcS-E9t, *rbcS-E9* gene terminator; gRNA1-Sc and gRNA2-Sc, guide RNA scaffold; zCas9, *Zea mays* codon-optimized *Cas9*; Hyg, hygromycin resistance gene. E, Characterization of edited *BnaA06.GTR2* transgenic lines. The upper panel shows gene structure and CRISPR–Cas9 target sites in *BnaA06.GTR2*. The Open boxes indicate 5'-untranslated region (5'-UTR) or 3′-UTR; the black boxes indicate exons; the black lines indicate the introns; The red boxes indicate sgRNA target sites. The lower panel shows the sequences of three T_0_ independent transgenic lines in the target sites. Deletions are indicated as blue hyphens. F, The phenotype of BnaA06.gtr2^CR-1^ T_2_ mutant during the vegetative (left) and reproductive (right) stages. G and H, Glucosinolate content of *BnaA06.GTR2* knockout mutants and the transgene-negative control (BnaA06.GTR2^WT^) in mature seeds (G) and developing seeds at 40 d after pollination (H). In (G) and (H), at least four plants per line were independently sampled for measurement. Each error bar is means ± sd (*n* ≥ 4 plants for each line). Student’s *t* test was used for statistical analysis, single asterisk indicates significant differences at *P *<* *0.05, double asterisk indicates significant differences at *P *<* *0.01, all compared to the BnaA06.GTR2^WT^.

To verify *BnaA06.GTR2* as a crucial player in seed glucosinolate accumulation and create variation for low seed glucosinolate breeding, we conducted clustered regularly interspaced short palindromic repeats/CRISPR-associated system 9 (CRISPR/Cas9) genome editing against *BnaA06.GTR2*. Two single-guide RNAs (sgRNAs) were designed to specifically target the third exon of *BnaA06.GTR2* (Figure 1, D and E; Supplemental Figure S2). Then the pHSE401-based vector Cas9-*BnaA06.GTR2* was transformed into ZY821 through *Agrobacterium tumefaciens*-mediated hypocotyl transformation (Xing et al., 2014), and 45 independent positive T_0_ transgenic plants were obtained. Among them, three plants (named BnaA06.gtr2^CR-1^, BnaA06.gtr2^CR-2^, and BnaA06.gtr2^CR-3^) in which homozygous small deletions occurred at both target sites for gRNA1 and gRNA2 (Figure 1E), produced three putative truncated proteins with respective sizes of 174, 168, and 173 amino acids. Then the three genome-edited plants were self-pollinated, and genome editing events were successfully transmitted to the T_1_ generation and no additional mutation was identified in the progenies of the three transgenic plants (Supplemental Table S3). High-performance liquid chromatography measurement showed a sharp reduction (76.05% ± 0.76% reduction on average) in total seed glucosinolate content with an average content of 32.6 ± 11.9, 31.5 ± 6.8, 30.6 ± 13.4 µmol·g^-1^ in the three transgenic lines, respectively, while the transgene-negative control (BnaA06.GTR2^WT^) was 131.8 ± 8.3 µmol·g^−1^ in mature seeds (Figure 1G; Supplemental Table S4; “Methods”). Predominant aliphatic glucosinolates existing in seed also showed significant reductions in three edited lines. The content of the indolic glucosinolates, accounting for a relatively small proportion in the total glucosinolate content, was not affected by *BnaA06.GTR2* knockout or even showed a slight increase in seeds. In developing seeds and silique walls at 40 d after pollination, the content of aliphatic, indolic, and total glucosinolates showed similar results as those in mature seeds (Figure 1H; Supplemental Figure S3 and Supplemental Table S4) although relative amount of aliphatic and indolic glucosinolates showed a slight difference from that of mature seeds. Furthermore, the loss-of-function mutants of *BnaA06.GTR2* showed no apparent alteration in morphological and yield-related traits (Figure 1F; Supplemental Figure S4 and Supplemental Table S5) in normal growth condition, and could serve as a good low seed glucosinolate germplasm. These results contrast with published data (Tan et al., 2022) that showed a ∼33.96% decline in seed glucosinolate content in a line (only one line) of CRISPR/Cas9*-*edited *BnaA06.GTR2* which was accompanied by seed weight decrease. The reason for the difference is probably due to the mutation difference: that line harbors one degenerate base of synonymous substitution at the 3′-end of coding sequence (CDS) of *BnaA06.GTR2*, but the three lines in the present study have small deletions resulting in three putative truncated proteins; unlike editing of multiple *BnaGTR2* genes (Tan et al. 2022), mutation of the major player of aliphatic glucosinolate transporter BnaA06.GTR2 did not have negative effect on yield-related traits, suggesting that *BnaA06.GTR2* may not be responsible for other trait formation.

In summary, we provided a method to identify functions of genes with rare or no genetic variation in natural populations, we used genome-editing to create a low seed glucosinolate resource that is rare in the germplasm, and we proved *BnaA06.GTR2* with rare natural mutation is crucial in seed glucosinolate accumulation in polyploid *B. napus*. The combination of our results and the published ones (Tan et al. 202) suggests that, probably due to subfunctionalization or neofunctionalization of these duplicated genes, simultaneous editing of polyploidy multiple duplicated genes or a gene family may not be an effective strategy for mutation of a target trait.

## Supplemental data

The following materials are available in the online version of this article.

##  

**Supplemental Figure S1.** Neighbor-joining tree of all GTRs homologs in *A.* *thaliana* and 12 *B.* *napus* genomes.

**Supplemental Figure S2.** Nucleotide sequence of *BnaA06.GTR2* in ZY821 genome.

**Supplemental Figure S3.** Glucosinolate content of *BnaA06.GTR2* knockout mutants and BnaA06.GTR2^WT^ control in developing silique walls at 40 d after pollination.

**Supplemental Figure S4.** Thousand-seed weight (g) in *BnaA06.GTR2* knockout mutants and BnaA06.GTR2^WT^ control.

**Supplemental Table S1.** Gene expression level of all *BnaGTRs* in 20 stages/tissues from ZY821 and ZS11.

**Supplemental Table S2.** The list of 312 *B. napus* germplasm accessions used for genome-wide association studies in this study.

**Supplemental Table S3.** Detection of mutations at potential CRISPR/Cas9 off-target sites in the T_1_ generation.

**Supplemental Table S4.** Glucosinolate content (μmol/g) in different tissues between *BnaA06.gtr2* mutants and their control in the T_2_ generation.

**Supplemental Table S5.** Thousand seed weight (g) of *BnaA06.gtr2* mutants and their control in the T_2_ generation.

**Supplemental Table S6.** Primers used in this study.

## Supplementary Material

kiac021_Supplementary_Data

## Notes

Y.Z., S.L., and Q.Y. designed the research. Y.H., Z.Y., and M.T. performed the experiments. Z.Y., Y.H., and M.T. analyzed the data. Y.H. and Y.Z. wrote the manuscript, and Y.Z. and S.L. revised the manuscript. All authors read and approved the final manuscript.

The author responsible for distribution of materials integral to the findings presented in this article in accordance with the policy described in the Instructions for Authors (https://academic.oup.com/plphys/pages/general-instructions) is: Yuanyuan Zhang (zhangyy@caas.cn).
