# Supplementary material for: Enhancing canola breeding by editing a glucosinolate transporter gene lacking natural variation
Source: Plant Physiol. 2022 Jan 25;188(4):1848–51. doi: 10.1093/plphys/kiac021 (PMC8968350; doi:10.1093/plphys/kiac021)
Supplement: kiac021_Supplementary_Data [file kiac021_supplementary_data.zip › Supplemental Data.pdf]

Supplemental Data

Supplemental Methods

Plant materials and growth conditions

All accessions in *B. napus* natural variation population (List in Supplemental Table S2) were planted at the experimental station of Oil Crops Research Institute of Chinese Academy of Agricultural Sciences (OCRI-CAAS), Yangluo (30°43'N and 114°31'E), in the growth seasons of 2016-2019. The transgenic materials were planted at the experimental station of OCRI-CAAS, Hanchuan (30°40'N and 113°43'E), in the growth seasons. Trial management followed standard breeding field protocols.

Constructing Neighbor-Joining tree

Protein sequences of BnaGTR1s, BnaGTR2s, BnaGTR3s, AtGTR1, AtGTR2, AtGTR3 and MeCGTR1 (*Manihot esculenta* cyanogenic glucoside transporter-1 as an out-group, Phytozome ID: Manes.15G180400) were aligned using Clustalw2 (v2.1) (Larkin et al., 2007). Then, Neighbor-Joining tree was constructed using MEGAX with 1,000 bootstrap replications (Kumar et al., 2018).

Plant tissue collection, RNA extraction and quantification for RNA-Seq

Transcriptional profiling of *BnaGTRs* from 20 tissues in two *B. napus* accessions with high glucosinolate (cv. ZY821) and low glucosinolate (cv. ZS11) content, respectively, was conducted as an RNA-seq experiment. In brief, tissues from seeds and silique walls at 4, 8, 12, 16, 20, 24, 28, 32, 40 and 48 days after pollination on main inflorescence in the two represented accessions were collected and immediately frozen by liquid nitrogen. Two biological replicates per sample and each replicate obtained from four independent plants, were pooled for transcriptome sequencing. Total RNA was extracted using the RNeasy Plant Mini Kit (QIAGEN) according to the manufacturer's instructions. Library of mRNA were prepared and sequenced using HiSeq 150-bp paired-end Illumina RNA-Seq protocol and produced ~384.48G data in total. The low-quality RNA-seq reads were firstly removed by Trimmomatic software (v0.36) (Bolger et al., 2014). Then, the clean data from accessions were mapped to ZY821 genome using Hisat2 (v2.1.2) with default parameters (Kim et al., 2015). Finally, Gene expression level (FPKM) was calculated using StringTie software (v1.3.5) with default settings (Pertea et al., 2015). All the raw sequencing data generated during this study are available in the database: http://yanglab.hzau.edu.cn/yls_ZY821_ZS11/rawdata/.

SNP calling

The genome re-sequencing data of 312 *B. napus* accessions were mapped to ZY821 reference genome respectively using BWA-MEM with default parameters (Li et al., 2013). Then, the reads with the mapping quality value < 10 were removed by SAMtools (v1.9) (Li et al., 2009). SNPs were identified using Sentieon DNAseq pipeline for all accessions. SNPs with low mapping quality were firstly filtered out by GATK VariantFiltration with parameters "QUAL < 30.0 || MQ < 50.0 || QD < 2" (McKenna et al., 2010). Then, the SNPs with minor allele frequencies (MAF) < 0.05 and missing rates > 0.1 were discarded by VCFtools (v 0.1.16) (Danecek et al., 2011). Genotype imputation was performed using beagle (v5.1) (Browning et al., 2018).

Genome-wide association study (GWAS)

The SNPs with a minor allele frequency (MAF) of less than 0.05 were filtered using VCFtools (Danecek et al., 2011), and finally 2,246,494 SNPs were left for genome-wide association study (GWAS). GWAS were performed for glucosinolate content using the EMMAX (Kang et al., 2010). The population structure analysis was performed by FastSTRUTURE (Raj et al., 2014). The population structure was controlled for EMMAX using results of K=3 as covariates, as well as a kinship matrix derived from all SNPs calculated by EMMAX. The cutoff for determining significant associations was set as 6.35 ($-\log_{10}(1/n)$), where n represents the total number of SNPs from 312 *B. napus* accessions. Then, five QTL loci located on chromosomes A02, A09, C02, C06 and C09 were significantly associated with seed glucosinolate content (Figure 1C) and three of them harboring five *BnaGTR2s* on chromosomes A02, A09 and C02. We didn't find any *BnaGTRs* in the QTL loci on chromosome C06 and C09. Maybe other glucosinolate biosynthesis genes in these regions contribute to seed glucosinolate accumulation, such as *BnaC06.GSTU20*, *BnaC09.MYB28* and *BnaC09.AP2* in these two QTL regions.

CRISPR/Cas9 plasmid construction, transformation and positive transgenic-plant identification

Two sgRNA target sequences specifically targeting the third exon region of *BnaGTR2.A06* were designed, respectively. CRISPR/Cas9 plasmid construction was conducted according to the method previously reported (Xing et al., 2014). In brief, to assemble two gRNAs, a single PCR fragment flanked by two sgRNA targets were amplified from pCBC-DT1T2 vector with

two pairs of partially overlapping primers, among which two forward and two reverse primers respectively contain one of the two target sites. Then the PCR fragment was purified and inserted into the binary vector pHSE401 by restriction-ligation reaction using *Bsa*I restriction enzyme and T4 Ligase (New England Biolabs).

The pHSE401-based CRISPR/Cas9 binary vector Cas9-*BnaA06.GTR2* verified by sequencing was transformed into *B. napus* cv. ZY821 through *Agrobacterium tumefaciens*-mediated hypocotyl transformation (Zhou et al., 2002). Genomic DNA from individual transgenic plants was extracted for PCR analysis. The PCR products containing the target sites were amplified with specific primers BnaGTR2.A06-pcr-F/ BnaGTR2.A06-pcr-R (Supplemental Table S6) from individual transgenic plants, and then cloned into pEASY-T3 vector (TransGen Biotech) for sequencing. The potential off-target sites were predicted and the six most likely off-target sites for each sgRNA in the transgenic plants were sequenced (Supplemental Table S3). Primers used in this study are listed in Supplemental Table S6.

Glucosinolate extraction and quantification

Silique wall and seed glucosinolate extraction and quantification were essentially performed according to previously described methods (Kliebenstein et al., 2001). For T_2 transgenic plants, we collected the seeds and silique walls at 40 days after pollination on the main inflorescence and immediately flash-frozen in liquid nitrogen. Then the samples after freeze dried were subjected to glucosinolate extraction. For mature seeds samples, the glucosinolate content in mature seeds of each line was measured after maturity, harvested and dried. The results were expressed as μmol/g dry samples. We used a transgene-negative ZY821 line BnaA06.GTR2^{WT} (transformed with the same construct, but without insertion of vector fragment) as the control for glucosinolate measurements, and we didn’t observe any significant phenotype changes (including glucosinolate content) between this transgene-negative line and wildtype ZY821 (without any treatment). We also detected glucosinolate content in several transgene-positive lines harboring inserted Cas9 coding sequence in which the targeted gene *BnaA06.GTR2* was not edited, and we found that there is no any obvious phenotype difference (including glucosinolate content) between the lines and either wildtype or transgene-negative ZY821 lines.

Supplemental References

Bolger AM, Lohse M, Usadel B (2014) Trimmomatic: a flexible trimmer for Illumina sequence data. Bioinformatics **30**: 2114-2120

Browning BL, Zhou Y, Browning SR (2018) A One-Penny Imputed Genome from Next-Generation Reference Panels. Am J Hum Genet **103**: 338-348

Danecek P, Auton A, Abecasis G, Albers CA, Banks E, DePristo MA, Handsaker RE, Lunter G, Marth GT, Sherry ST, et al. (2011) The variant call format and VCFtools. Bioinformatics **27**: 2156-2158

Kang HM, Sul JH, Service SK, Zaitlen NA, Kong SY, Freimer NB, Sabatti C, Eskin E (2010) Variance component model to account for sample structure in genome-wide association studies. Nat Genet **42**: 348-354

Kim D, Langmead B, Salzberg SL (2015) HISAT: a fast spliced aligner with low memory requirements. Nat Methods **12**: 357-360

Kliebenstein DJ, Kroymann J, Brown P, Figuth A, Pedersen D, Gershenzon J, Mitchell-Olds T (2001) Genetic control of natural variation in *Arabidopsis* glucosinolate accumulation. Plant Physiol **126**: 811-825

Kumar S, Stecher G, Li M, Knyaz C, Tamura K (2018) MEGA X: Molecular Evolutionary Genetics Analysis across Computing Platforms. Mol Biol Evol **35**: 1547-1549

Larkin MA, Blackshields G, Brown NP, Chenna R, McGettigan PA, McWilliam H, Valentin F, Wallace IM, Wilm A, Lopez R, et al. (2007) Clustal W and Clustal X version 2.0. Bioinformatics **23**: 2947-2948

Li, H (2013) Aligning sequence reads, clone sequences and assembly contigs with BWA-MEM. ArXiv **1303**

Li H, Handsaker B, Wysoker A, Fennell T, Ruan J, Homer N, Marth G, Abecasis G, Durbin R (2009) The Sequence Alignment/Map format and SAMtools. Bioinformatics **25**: 2078-2079

Zhou Y, Wang H, Gilmer S, Whitwill S, Keller W, Fowke LC (2002) Control of petal and pollen development by the plant cyclin-dependent kinase inhibitor ICK1 in transgenic *Brassica* plants. Planta **215**: 248-257

McKenna A, Hanna M, Banks E, Sivachenko A, Cibulskis K, Kernytsky A, Garimella K, Altshuler D, Gabriel S, Daly M, et al. (2010) The Genome Analysis Toolkit: a MapReduce framework for analyzing next-generation DNA sequencing data. Genome Res **20**: 1297-1303

Pertea M, Pertea GM, Antonescu CM, Chang TC, Mendell JT, Salzberg SL (2015) StringTie enables improved reconstruction of a transcriptome from RNA-seq reads. Nat Biotechnol **33**: 290-295

Raj A, Stephens M, Pritchard JK (2014) fastSTRUCTURE: variational inference of

population structure in large SNP data sets. Genetics **197**: 573-589

Xing HL, Dong L, Wang ZP, Zhang HY, Han CY, Liu B, Wang XC, Chen QJ (2014) A CRISPR/Cas9 toolkit for multiplex genome editing in plants. BMC Plant Biol **14**: 327

Supplemental Figures

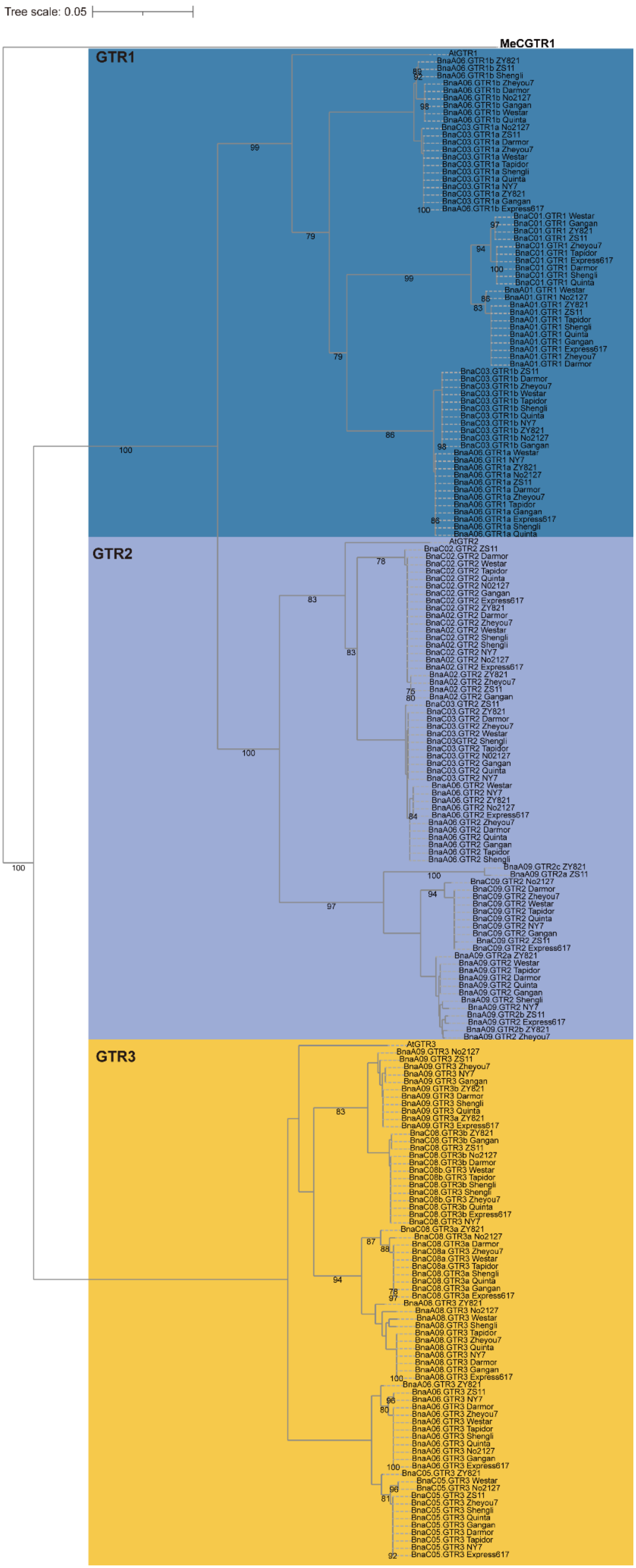

Supplemental Figure S1. Neighbor-joining tree of all GTRs homologs in *Arabidopsis thaliana* and 12 *Brassica napus* genomes. The phylogenetic tree was constructed using MEGAX. Members in the same clade are marked by the same color. Bootstrap values above 75% were given at branch nodes. Dotted lines indicate the terminal branches corresponding to the right-justified labels. MeCGTR1 (*Manihot esculenta* cyanogenic glucoside transporter-1) was used as an out-group. The scale bar indicates the number of amino acid residue substitutions per site.

ATGGAGAGAAAGCCTCTAGAAGTCGAGTCAACGGATCACCAAAAGCCTCCCTCCGCCGTGTACGATGGCTCTGTTAT
GGCGGTTGATTCTGTTGAGGAAGAAGTTGAGGAGACCAAAGTCGTTTACAGAGGCTGGAAAGTCATGCCCTTTATCAT
TGGTAACACAAACTTTTTTTTTATAAACTTTCTTCTTTTGTTCCAAGAACTTTTTTTTTTAATTGCTTTTCATGTTTTTTTC
CAAAATAAATAATCAATAGTTGAAGAAATGAAGAGGAAAACATCAGTCTTGCTTGAAGACTTATTGGTTTGATTTAA
AAAAAAAAAATCGTGAGATTCTCTGTACTGTTGTCAAAAGCTTTGACTTAGTCAACCAGTTTTTTAAGAAAATTAAAA
AAAAGAGAGTGAGATCTAGAATTTGAGAAGGTTTCAGAACATAACTTTGAGGCATATGAGATCAAAGTCTTATGATC
ATCTTGGTTTTGTTTGTTGGTATTATCAGGAAATGAGACGTTTGAGAAGCTTGGGATCATTGGAACACTATCAAACCTT
CTGGTTTATTTAACTGCTGTCTTCAACATGAAGAGTATCACAGCTGCAACAATCATCAATGCATTCAGTGGCACAATC
AACTTCGGAACCTTCGTTGCTGCTTTCCTCTGCGACACTTACTTCGGTCGATACAAGACTCTAACTGTCGCTGTCATCG
CCTGTTTTCTTGTAATGATCATTCCTTTGCAATCTTTCTTGCTCCACAAGCGTTCTTAGTTTAACAACCCTAAAAATCTG
ATAATTGTTGTTCTTTTTTTTTTGTAGGGATCACTTGTGATACTATTGACAGCTGCAGTGCCACAATTACATCCAGCTCC
ATGCGGAACAGCTCTCTCATGTATCGGGCCAAGTGGTGGCCAGATAGCGTTTCTACTGCTGGGTCTCGGGTTTCTTGT
AGTTGGAGCAGGCGGGATTAGACCATGTAATCTAGCTTTCGGGGCTGATCAGTTTAACCCCAAGAGCGAGTCAGGAA
AACGAGGCATCGATAGTTTCTTCAATTGGTACTTCTTCACCTTCACGTTCGCGCAGATCCTGTCGCTGACACTAATCGT
CTATATCCAGTCCAATGTCAGCTGGACAATCGGTTTAACCATCCCGGTCGTTCTCATGTTCTTGGCCTGCGTGATTTTC
TTTGCTGGAGATAAGTTGTATGTGAAGATCAAAGCCTCAGGTAGTCCATTGGCTGGTATAGCTCAAGTTATAGCGGTT
GCAATCAAGAAACGTGGGTTAAAGCCAACGAAAGAGCCTTGGCTTAACCTTTACAACTACTACCCACCAAAATACGC
AAACTCCAAACTCAAATACACCGACCAGTTTAGGTAACAAAACACACACACGCCAAGAGTTAGTTTTATATTTAATAT
CATATCTTTCCACTTTTGTTTTATGGTTACTGAGGAAGTTTCCTGTTTCGGCAGGTTCCTTGATAAGGCGGCGATTTTGG
CTCCCGAAGACAAGTTGGAGGCTGATGGTAAGCCTGCGGATCCCTGGAAGCTTTGTACAATGCAACAAGTTGAAGAA
GTGAAGTGCATTGTGAGAGTGCTTCCTATATGGTTTGCTGCATCGATCTATTACTTGACCATCACGCAACAAATGACTT
ACCCCGTCTTCCAAGCCCTCCAGAGCGACCGTCGCTTAGGATCCAGAGGATTTGTGATCCCGGCAGCCACCTACGTTG
TCTTTTTGATGACAGGAATGACGGTTTTCATCATATTCTACGACCGTGTCCTCGTGCCTACCTTAAGAAGAATAACCGG
TATAGACACGGGGATAACGCTACTGCAGAGGATTGGAACCGGAATTTTCTTCGCCTTTGTTAGCTTGATAGTCTCCGG
TTTCGTCGAGGAACGGAGGAGAACGTTCGCGCTGACTAAACCAACACTTGGTATGGCTCCAAGGAAGGGAGAAATCT
CCTCAATGTCAGCTATGTGGCTGATCCCACAACTCGCCCTTGCGGGTATAGCTGAGGCGTTTGGAGCTATAGGACAGA
TGGAGTTTTACTACAAGCAGTTCCCTGAGAACATGAGGAGTTTTGCTGGTTCGATCTTCTATGTAGGAGCAGGAGTTT
CGAGTTACCTCGGTAGCTTCTTGATTGCAATGGTTCACAGGACGACGCAGAACTCAGCTGGTGGTAATTGGTTGGCTG
AGGATTTGAACAAAGGAAGATTGGATTACTTCTATTTCATGATCGCTGGAATCTTGGCAGTTAATTTTGCTTACTTCTT
GGTCATGTCAAGATGGTATAGATACAAAGGAAGTGATGGTGAAGTGACAACTTATGAAACCAATGGAGATGCCATCA
AACAACAAGACAAGAATACTGTCTGA

Supplemental Figure S2. Nucleotide sequence of *BnaA06.GTR2* in ZY821 genome. The primers used for amplifying and sequencing targeted fragments of *BnaA06.GTR2* are indicated in green. Single-guide RNAs (sgRNAs) are highlighted in orange, followed by protospacer associated motifs (PAMs) that are shown in blue.

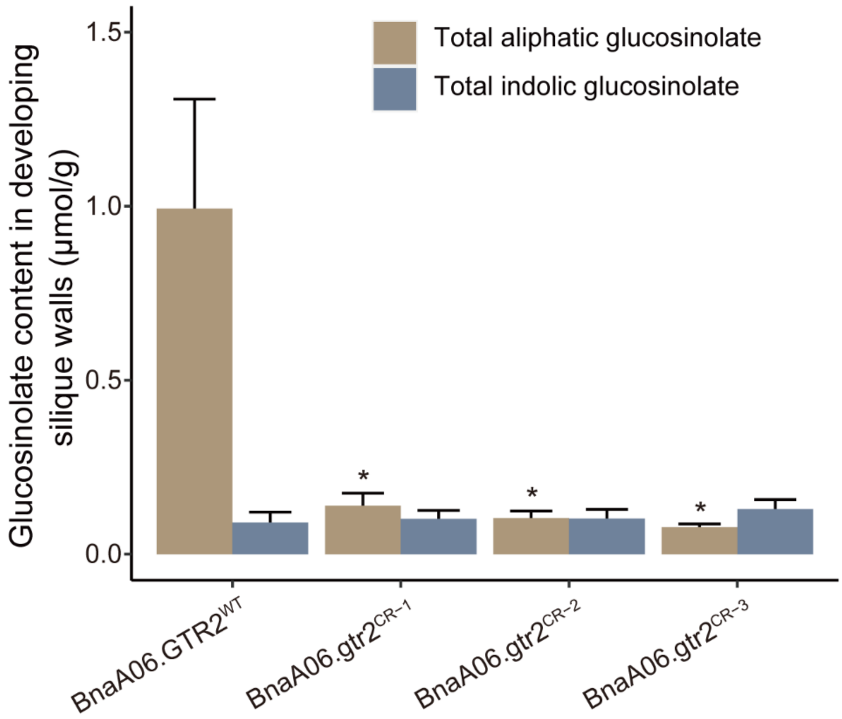

Supplemental Figure S3. Glucosinolate content of *BnaA06.GTR2* knockout mutants and BnaA06.GTR2^{WT} control in developing silique walls at 40 days after pollination. At least four plants per line were independently sampled for measurement. Each error bar is means ± SD (n ≥ 4 plants for each line). Student's t-test was used for statistical analysis, * indicates significant differences at $P < 0.05$, all compared to the BnaA06.GTR2^{WT} control.

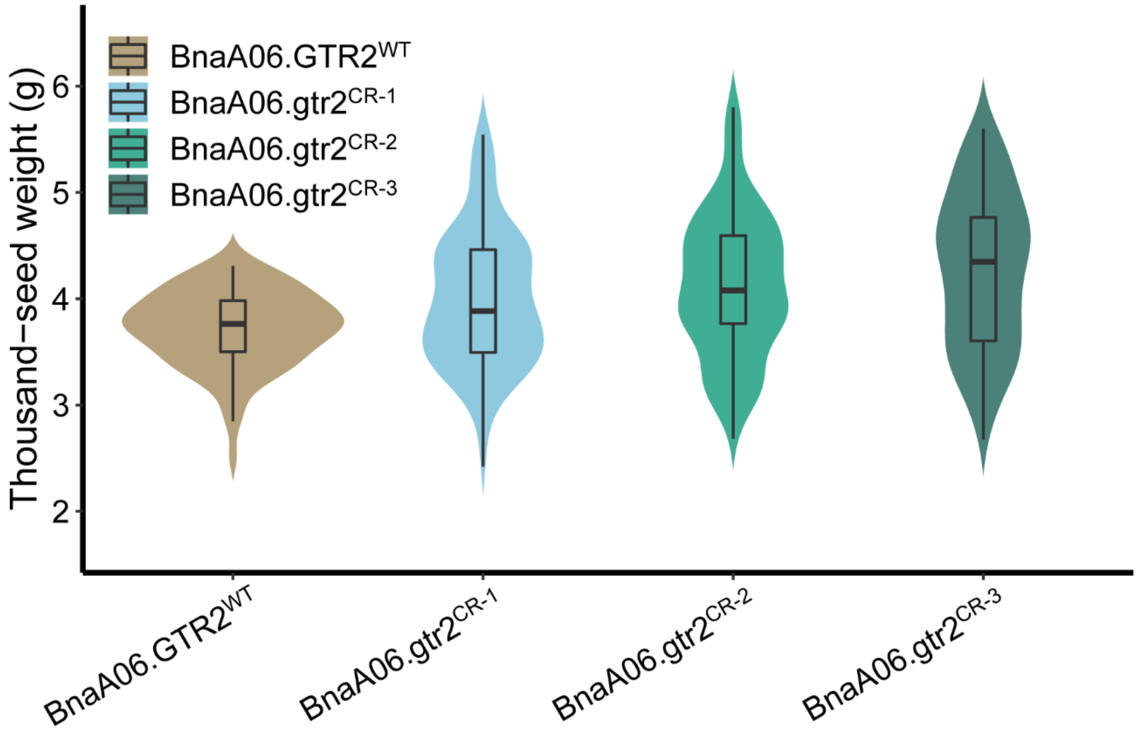

Supplemental Figure S4. Thousand-seed weight (g) in *BnaA06.GTR2* knockout mutants and BnaA06.GTR2^{WT} control. Each color represents a transgenic line. The violin plots show the distribution density and box plots show the distribution quantiles. The middle bars represent the medians, while the bottom whiskers, bottom edges of the boxes, top edges of the boxes, and top whiskers indicate the 5th, 25th, 75th, and 95th percentiles, respectively. At least sixty plants per line were sampled for measurement.
